# Human Microbiome and Its Medical Applications

**DOI:** 10.3389/fmolb.2021.703585

**Published:** 2022-01-13

**Authors:** Yangming Zhang, Linguang Zhou, Jialin Xia, Ce Dong, Xiaozhou Luo

**Affiliations:** ^1^ CAS Key Laboratory of Quantitative Engineering Biology, Shenzhen Institute of Synthetic Biology, Shenzhen Institute of Advanced Technology, Chinese Academy of Sciences, Shenzhen, China; ^2^ Department of Pharmacy, Peking University International Hospital, Beijing, China; ^3^ Department of Physiology and Pathophysiology, School of Basic Medical Sciences, Peking University, Key Laboratory of Molecular Cardiovascular Science, Ministry of Education, Beijing, China

**Keywords:** commensal microbiome, engineered microbe, synthetic biology, probiotics, medical application

## Abstract

The commensal microbiome is essential for human health and is involved in many processes in the human body, such as the metabolism process and immune system activation. Emerging evidence implies that specific changes in the microbiome participate in the development of various diseases, including diabetes, liver diseases, tumors, and pathogen infections. Thus, intervention on the microbiome is becoming a novel and effective method to treat such diseases. Synthetic biology empowers researchers to create strains with unique and complex functions, making the use of engineered microbes for clinical applications attainable. The aim of this review is to summarize recent advances about the roles of the microbiome in certain diseases and the underlying mechanisms, as well as the use of engineered microbes in the prevention, detection, and treatment of various diseases.

## Introduction

Many studies have highlighted that the commensal microorganism plays an important role in the development of human diseases. Thus, the term “holobiont” is used to describe the complex network and system formed by human cells and the commensal microorganism (Bordenstein and Theis, 2015; Inda et al., 2019). The microbiome is used to describe all microorganisms and their genomes, including bacteria, archaea, viruses, and fungi. Commensal microbiomes, such as the gut microbiome, skin microbiome, and vagina microbiome, contain beneficial microbes and pathogens, which contribute to host homeostasis in different locations. Therefore, modulating the microbiome is regarded to be an effective way to regulate host homeostasis and defeat diseases.

The earliest research on using bacteria to treat disease dates back to 1891, when Willian Coley invented Coley’s Toxins to treat cancer (Coley, 1891). In 1974, Parker introduced the term “probiotics” similar to the current definition (Parker, 1974). Probiotics are defined as live microorganisms that, when administered in adequate amounts, confer a health benefit on the host, such as several species from *Lactobacillus*, *Bifidobacterium,* and *Streptococcus* (Reid et al., 2003; Mays and Nair, 2018; Swanson et al., 2020; Zuo et al., 2020). However, some studies have shown the risks of adverse effects and ineffectiveness of probiotic use, indicating that more research is needed for a predictable and effective use of probiotics in medical applications (Costeloe et al., 2016; Freedman et al., 2018; Schnadower et al., 2018; Hill, 2020).

As of April 26, 2021, there are 5,416 studies related to the microbiome (searched by microbiota OR microbiome OR probiotic) registered on clinicaltrails.gov, of which 2,212 have been completed. European countries and the United States have the majority of the cases, followed by China, Canada, and India. Japan seems to have the least number of microbiome clinical trials out of the developed countries. These studies mainly applied unmodified microbes on metabolic diseases, tumors, infections, and other immune system diseases, whereas engineered microbes were used on tumors (Supplementary Table S1).

Synthetic biology is dedicated to understand, design, and modify natural lives to support human development. Because of the widely reported relevance of the microbiome and host health, engineering microbes for medical usage has become an emerging research direction (Bober et al., 2018; Inda et al., 2019). Their applications include modulating host metabolism, regulating the host immune system, combating pathogens, as a novel diagnosis tool or sensor, and as tools for host microbiome relationship discovery. With the development of more synthetic biology tools, the obstacles along the way for effective therapy with engineered microbes have been gradually removed (Goh and Barrangou, 2019; Liao et al., 2019; Naydich et al., 2019; Pedrolli et al., 2019; Buss et al., 2021). In this review, we will present an update on the study of commensal microbiome-host interactions, and summarize the use of probiotics or engineered microbes in the treatment of various diseases.

## Microbiome and Metabolic Diseases

Metabolic diseases are a set of diseases which are due to the disruption of normal metabolism, such as obesity, diabetes, non-alcohol fatty liver disease (NAFLD), and hyperuricemia. In most circumstances, metabolic diseases are the result of the combination of metabolism pathway abnormalities and escalated inflammation. A plethora of data accumulated in the past decades has tightly linked metabolic diseases with the commensal microbiome, suggesting the commensal microbiome is involved in host metabolism abnormalities and inflammation via several mechanisms. Intervention on the microbiome including probiotics, FMT, or engineered microbes could regulate host-microbiome interactions and provide beneficial outcomes for metabolic diseases.

### The Microbiome Impacts Host Metabolism

**FIGURE 1:**
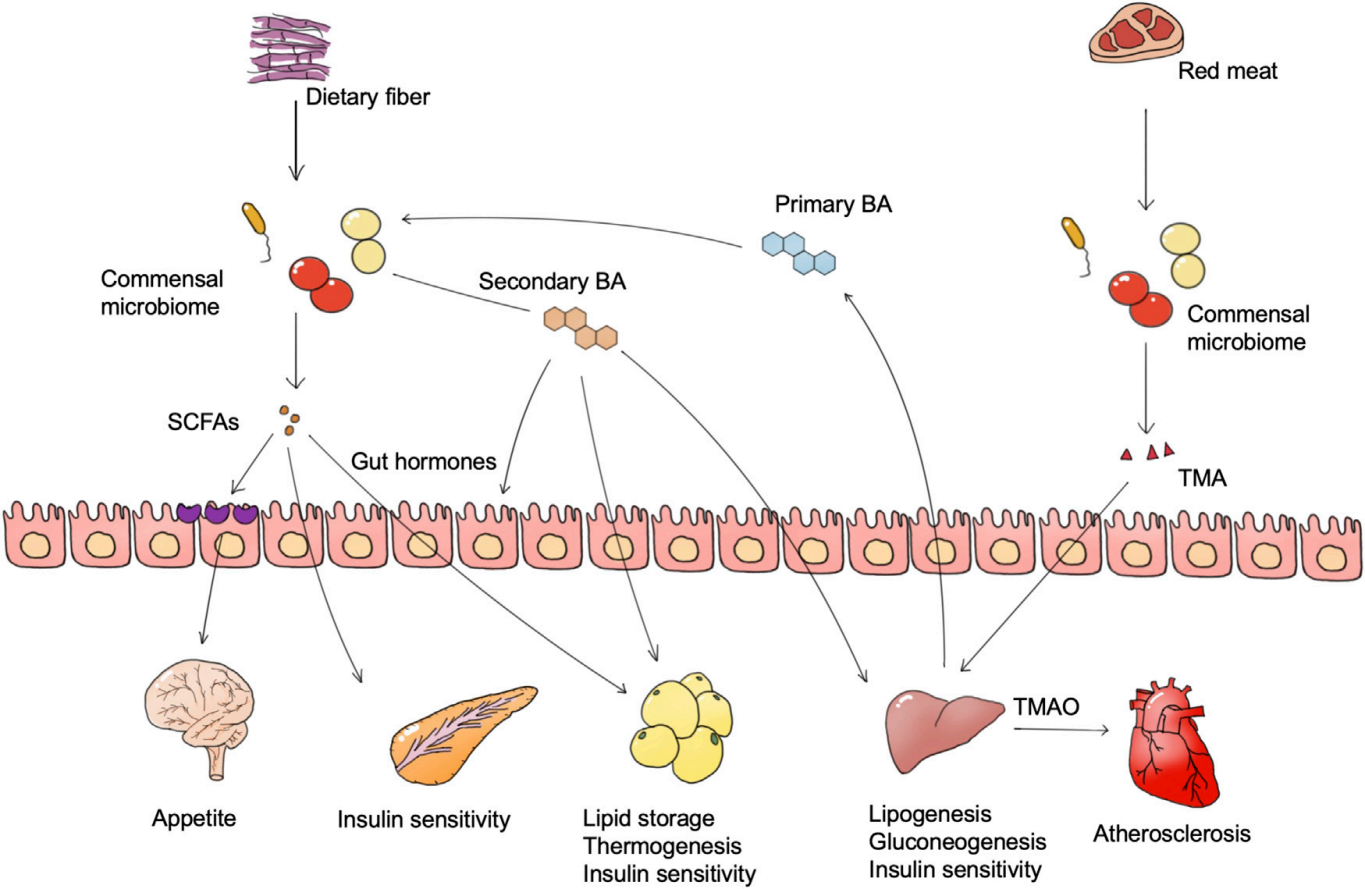
Microbiome-derived metabolites and metabolic diseases. Foods such as dietary fiber and red meat are degraded by the microbiome to generate multiple bioactive substances, of which SCFAs and TMAO are widely confirmed as important. The microbiome also converts host bile acids into more complex bile acid species. These microbiome-derived metabolites regulate the metabolism pathway in different tissues, and are finally involved in metabolic abnormalities of metabolic diseases. Abbreviations: SCFAs, short-chain fatty acids. BA, bile acid. TMA, trimethylamine. TMAO, trimethylamine oxide.

The most direct way for the commensal microbiome to participate in host metabolism is through the metabolites secreted by them which can regulate the metabolic processes of the host. In this process, the intestinal microbiome plays a key role. The gut microbiome provides extra nutrition for the host, including the degradation of protein and glycosaminoglycans and the production of short-chain fatty acids (SCFAs), bile acids, amino acids, and vitamins. In addition, the gut microbiome generates various enzymes, primary and secondary metabolites, and together affecting host metabolism processes (Canfora et al., 2015; Chen S et al., 2019; Zhang et al., 2019a; Wu J et al., 2021; Wu et al., 2021a). Among these microbiome-derived metabolites, SCFAs, bile acids, and trimethylamine (TMA) are considered to have the most impact on metabolic diseases (Figure 1).

#### SCFA

SCFAs, including acetate, propionate, and butyrate are proved to have both positive and negative influences on obesity. The gut microbiome degrades dietary fiber and monosaccharides into SCFAs, and different fiber types facilitate the production of different types of SCFAs (Woting and Blaut, 2016; Deehan et al., 2020). SCFAs are absorbed by intestinal epithelial cells as an energy source, and eventually reach the blood, affecting metabolism in muscle, liver, and fat. Obesity is characterized as adipose cell proliferation and hypertrophy. Oral intake of butyrate in mice could directly activate thermogenesis in brown adipose tissue to increase energy expenditure and reduce body weight (Gao et al., 2009). Propionate and butyrate also exhibit an anti-obesity effect via the stimulation of anorexigenic hormones and leptin synthesis, which is partly dependent on the combination of GPR41 (Al-Lahham et al., 2010; Lin et al., 2012; Nishida et al., 2021). Besides, SCFAs could stimulate glucagon-like peptide-1 (GLP-1) and peptide YY (PYY) hormones secretion and decrease appetite via the gut-brain axis (Tolhurst et al., 2012; Nishida et al., 2021). However, in a human study, the increase of SCFA producers such as *Eubacterium ventriosum* and *Roseburia intestinalis* is positively associated with obesity, while the abundance of butyrate producer *Oscillospira* spp. is positively related to leanness (Tims et al., 2013; Gophna et al., 2017). One reason may be that the excess SCFAs act as a direct energy source to increase energy intake. Another reason may due to the functions of acetate. Acetate is a direct substance for lipogenesis, which may contribute to adiposity, and a mice study suggested that microbiome-derived acetate production can activate the parasympathetic nervous system and lead to hyperphagia and obesity (Perry et al., 2016).

Current evidence from rodent and human studies also implicates microbiome-derived SCFAs in glucose metabolism regulation where different SCFAs have different functions. Over 95% of diabetes in adults is type II diabetes (T2D), which is characterized by insulin resistance and hyperglycemia. Nutritional fiber intervention of T2D patients has enriched a functional group of 15 strains of acetic acid and butyric acid-producing bacteria such as *Faecalibacterium prausnitzii*, *Eubacterium rectale,* and *Ruminococcus* sp.*,* and inhibited those strains capable of producing indole and hydrogen sulfide, leading to an increased butyric acid level which promoted glucagon-like peptide-1 (GLP-1) and peptide YY (PYY) secretion to elevate blood insulin level. The abundance and diversity of the enriched strains were highly positively correlated with diabetes therapy outcomes (Zhao et al., 2018). Two cohorts in south China also demonstrated that people with high fruit intake exhibited lower T2D risk. High fruit intake accumulated *Faecalibacterium prausnitzii*, *Akkermansia muciniphila*, *Ruminococcaceae*, *Clostridiales*, and *Acidaminococcus* in the gut microbiome, which may benefit insulin sensitivity through increasing production of SCFAs (Jiang et al., 2020). However, it is still controversial that the presence of some types of SCFAs could benefit T2D. Sanna et al. (2019) constructed a computational model with genome information of the gut microbiome and their human hosts as well as metabolic traits of hosts to find associations between the gut microbiome and host metabolism. The results showed that an increase in intestinal butyrate produced by butyrate-producing bacteria such as *Eubacterium rectale* and *Roseburia intestinalis* could improve host insulin response, whereas abnormal propionate production and absorption would elevate intestinal propionate level, leading to a higher risk of type 2 diabetes. Tirosh et al. (2019) reported that propionate intake stimulated glucagon and fatty acid-binding protein 4 (FABP4) production, thus inducing insulin resistance in mice and humans (Tirosh et al., 2019). Together, these findings support an important role for microbiome-derived SCFAs, in which butyric acid may be metabolically protective while the function of propionic acid is still unclear.

#### Bile Acid

The gut microbiome generates bile acids to regulate host metabolic processes (e.g., glucose, lipids, and energy homeostasis) via bile acid receptors (e.g., FXR and TGR5), and the regulation outcomes differ among tissues (Fan and Pedersen, 2021). The gut microbiome generates various secondary bile acids by metabolizing primary bile acids through deconjugation and dihydroxylation, and different types of bile acids have distinct influence on host metabolism (Wahlstrom et al., 2016). Hypothalamic TGR5 activation is regarded as an anti-obesity process. Supplement of a bile acid mixture (taurocholic, glycocholic, deoxycholic, and cholic acid) exhibited an anti-obesity effect via the activation of hypothalamic TGR5 (Castellanos-Jankiewicz et al., 2021). In terms of mechanism, hypothalamic TGR5 activation on the AgRP neuron, instead of the POMC neuron, decreases orexigenic agouti-related peptide/neuropeptide Y (AgRP/NPY) release, and thus increases satiety and reduces food intake (Perino et al., 2021).

Intestinal FXR activation is reported to be crucial in microbiome dysbiosis-induced obesity (Parséus et al., 2017). Inhibition of intestinal FXR via glycoursodeoxycholic acid (GUDCA) improved mice obesity, whereas deconjugation of GUDCA by *Bacteroides fragilis* in the gut led to glucose intolerance and abrogated the metabolic benefits of metformin treatment (Sun et al., 2018). The activation of FXR in the gut, however, was beneficial for lipid metabolism as it could inhibit lipid uptake and ameliorate NAFLD (FXR activation protects against NAFLD via bile-acid-dependent reductions in lipid absorption). Oral intake of lithocholic acid (LCA) and ursodeoxycholic acid (UDCA) activated the FXR pathway and repaired gut barrier integrity, leading to a reduction of hyperlipidemia. It was thought that intestinal FXR activation can impair glucose metabolism and improve lipid metabolism. However, selective activation of intestinal FXR increased brown adipose tissue thermogenesis and insulin sensitivity, and also reduced body weight. The conflicting results may be due to the selective activation of GPCR, thus more research is needed to discover the detailed function of every bile acid species *in vivo*.

Unlike FXR, activation of gut and adipose TGR5 could be anti-diabetes and anti-obesity processes. Internal production of cholic acid-7-sulfate (CA7S) activated gut TGR5, and thus stimulated GLP-1 and insulin production, leading to a decrease of blood glucose level (Chaudhari et al., 2021a). Hyocholic acid (HCA) activated intestinal L cells via TGR5 signaling and FXR signaling, promoting GLP-1 production and secretion to enhance insulin sensitivity and lower blood glucose. Reduced serum HCA concentrations were associated with elevated markers of diabetes and glucose in a clinical cohort. Recently, a detailed mechanism study reported that bile acid level and host metabolism were regulated by complex host-microbiome interaction. Intestinal hypoxia-inducible factor 2α (HIF-2α) increased lactate levels in the intestine through upregulation of lactate dehydrogenase A expression, and lactate enhanced polysaccharide utilization leading to the growth of *Bacteroides vulgatus*. The growth of *Bacteroides vulgatus* inhibited the growth of *Ruminococcus torques*, which was able to generate taurocholic acid (TCA) and deoxycholic acid (DCA). TCA and DCA upregulated UCP1 and CKMT2 expression by activating the bile acid receptor TGR5 in adipocytes, thereby promoting white adipose tissue thermogenesis and ameliorating obesity in mice. Supplementation of mice with TCA or DCA and gavage of *Ruminococcus torques* ameliorated insulin resistance and obesity (Wu et al., 2021b). This study not only elucidates the role of the microbiome and their bile acid products in bridging host genes and host metabolic regulation, but also provides a good paradigm reference for subsequent studies of the microbiome on metabolic diseases. The diversity of bile acid enzymes produced by the gut microbiome leads to the production of a pool of diverse bile acids, the exact mechanism of which needs to be confirmed under various physiological conditions and disease models.

#### TMAO

TMAO is an important biomarker for atherosclerosis and diabetes. Dietary quaternary amines (mainly phosphatidylcholine, lecithin, and L-carnitine) can be converted into TMA by the gut microbiome and further to trimethylamine oxide (TMAO) in the liver (Hoyles et al., 2018). Plasma TMAO levels correlated with plaque instability characteristics such as inflammation, platelet activation, and intraplaque hemorrhage, and is regarded to be positively correlated with the risk of atherosclerosis and cardiovascular events (Koay et al., 2021). In clinical cohorts, a dose-dependent positive correlation between circulating TMAO levels and diabetes risk was likewise found, and each 5 μmol/L increase in plasma TMAO was associated with a 54% increase in diabetes prevalence (Zhuang et al., 2019). It has been reported that TMAO production in insulin resistance leads to the activation of endoplasmic reticulum stress kinase PERK and the corresponding unfolded protein response and promoting hyperglycemia (Chen S et al., 2019).

In addition to regulators such as SCFA, bile acids, and TMAO, the gut microbiome can also be involved in host metabolite exchanges, such as purine, fatty acids, and glucose, which may also lead to certain metabolic diseases. These direct or indirect linkages between the gut microbiome and host metabolism give us opportunities to manage metabolic diseases through the manipulation of the microbiome.

### The Microbiome Influences Host Inflammation Level

Another important way which the gut microbiome influences metabolic diseases is via the regulation on the immune system. A low-grade inflammation level is reported to commonly exist in metabolic diseases. Low-grade systemic inflammation is identified in many metabolic diseases including obesity, T2D, and NAFLD (Tran et al., 2020). Unlike the acute inflammation response in infection or injury, inflammation in metabolic diseases is usually chronic, and the main organs affected by inflammation include adipose tissue, the liver, and cardiovascular system. Eran Elinav has proposed a compelling paradigm in which a “gastrointestinal hit” such as dysbiosis and intestinal barrier dysfunction could be a promoter in metabolic diseases (Tilg et al., 2020). Thus, the inflammation induced by the disturbed microbiome could be another major promoter for some metabolic diseases (Fan and Pedersen, 2021).

The disruption of the intestinal barrier is an important trigger of metabolic inflammation. The intestinal barrier is composed of the intestinal epithelium, mucus layer, local immune system, and their secretion which is important to maintain gut homeostasis. The segregation of the intestinal epithelium and microbiome is maintained by the mucus layer and a moderate immune response. Intestinal barrier dysfunction results in the translocation of the microbiome and pathogen-associated molecular pattern molecules (PAMPs), for example, lipopolysaccharides (LPSs), and thus stimulates immune system activation and leads to an increase in inflammation. It is already known that in some metabolic diseases, such as obesity, T2D, and NASH, the intestinal barrier is disrupted and intestinal permeability is increased (Cani, 2016; Tilg et al., 2020). In some obese individuals and T2D patients, bacteria were found in the blood and a variety of adipose tissues, which is believed to come primarily from the intestine and has an important role in triggering and maintaining adipose tissue inflammation (Anhe et al., 2020; Massier et al., 2020). The presence of a normal gut microbiome is crucial to mucus layer secretion which forms the first defensing barrier, whereas germ-free mice exhibit lower goblet cell numbers and a thinner mucus layer (Paone and Cani, 2020). Production of endotoxin or several microbial products stimulates mucus layer secretion and is regarded as a behavior of defense. However, in dysbiosis, some mucus-degrading species (e.g., *Escherichia histolytica*) proliferation and chronic inflammation induce a depletion of goblet cells and disrupt the intestinal barrier. In metabolic diseases, metabolism challenges such as HFD leads to dysbiosis, stimulating a pro-inflammatory signaling cascade response that increases barrier-degrading cytokines (TNF-α, IL-1β, IL-6, and IFN-γ) and decreases barrier-forming cytokines (IL-10, IL-17, and IL-22), thereby disrupting the intestinal barrier and enhancing intestinal permeability (Christ and Latz, 2019). Intervention on dysbiosis including microbiome depletion or immune response inhibition reduces pro-inflammatory cytokines secretion and ameliorates remote inflammation, suggesting a stable microbiome-intestinal barrier is important in gut homeostasis and host metabolism (Tran et al., 2020).

Microbiome-derived metabolites could regulate immune system response and metabolic inflammation level, which is another important mechanism in metabolic inflammation. Long-chain fatty acids are reported to have a pro-inflammation effect, in contrast, SCFAs are usually regarded as anti-inflammatory microbiome-derived metabolites (Haghikia et al., 2016). SCFAs regulate immune response and inflammation by reducing the recruitment and migration of macrophages, neutrophils, and dendritic cells and inhibiting the differentiation of T cells and B cells, via different SCFA receptors such as GPR41, GPR43, and GPR109A. Addition of dietary fiber intake is proved to ameliorate inflammation in obesity, T2D, NAFLD, and cardiovascular diseases via increased production of SCFAs. Bile acids are another major class of immunomodulatory substances secreted by the intestine microbiome via a receptor including TGR5 and FXR. Hydrophobic bile acids such as LCA, DCA, and CDCA are thought to be cytotoxic, inducing the accumulation of ROS and the elevation of the inflammatory factor TNF-α, whereas hydrophilic bile acids seem to be anti-inflammatory. For example, deconjugation of a hydrophilic glycodeoxycholic acid (GDCA) by *Bacteroides vulgatus* in the gut led to a reduction of IL-22 production in innate lymphoid cells (ILC3s), which aggravated insulin resistance in a mice model of polycystic ovary syndrome (Qi et al., 2019). Some other microbiome-derived metabolites also exhibit an immune system regulation effect. Yuan et al. (Yuan et al., 2019) reported a prevalence of high alcohol-producing *Klebsiella pneumoniae* in NAFLD patients. *K. pneumoniae* produces large amounts of alcohol, and the endogenous alcohol produced by these bacteria is an important causative agent of inflammation and NAFLD.

In most circumstances, metabolic diseases are the result of the combination of metabolism pathway abnormalities and inflammation. For example, it has been shown that HFD promoted the growth of the pathogenic commensal bacteria *Bilophila wadsworthia*, which promoted lipopolysaccharide (LPS) production and reduced butyric acid production. These changes not only exacerbated HFD-induced inflammation and intestinal barrier dysfunction, but also impaired bile acid metabolism and induced glucose metabolism disorders and fatty liver. Supplementation with *Lactobacillus rhamnosus* CNCM I-3690 reduced *B. wadsworthia*-induced inflammation and metabolic damages in mice by limiting its growth (Natividad et al., 2018). Nonalcoholic steatohepatitis (NASH) is another recognized example indicating that metabolic diseases result from a combination of metabolism abnormalities and inflammation. The pathogenesis of NASH is regard as a two-step hit process. The first hit is the lipid accumulation in the liver, and the second hit is the inflammation progress. In the first hit, the gut microbiome converts fructose to acetic acid, which is converted to acetyl coenzyme A by the enzyme ACSS2 in the liver, promoting *de novo* lipogenesis (Zhao Q et al., 2020). Besides, the gut microbiota class *Collinsella* is reported to be enriched in NASH patients, and is positively correlated with the increase of fasting levels of triglycerides and total cholesterol (Astbury et al., 2020). In the second hit, gut microbiome disorder promotes inflammation via driving disruption of the gut vascular barrier and translocation of bacteria to the liver (Mouries et al., 2019). In summary, metabolic disease is the result of the combination of metabolism abnormalities and inflammation, while microbiome disorder is involved to promote metabolic diseases.

### Microbiome Intervention and Engineered Microbe Application

Microbiome intervention includes untargeted methods (e.g., antibiotics, exercises, probiotics, dietary nutrition, and fecal microbiota transplantation [FMT]) and targeted methods (e.g., engineered microbes and targeted drugs). Owing to the important role of the microbiome in metabolic disease, it is promising to treat host metabolic disorders with a microbiome intervention method.

FMT is one promising strategy to alter the gut microbiome in metabolic diseases (Juul et al., 2018). Patients with metabolic syndrome that received FMT from lean donors (allogenic group) experienced an improved insulin sensitivity compared to the autologous FMT group 6 weeks after the transplantation, which was attributed to the beneficial butyrate-producing microbiota *Roseburia intestinalis* and *Eubacterium hallii* from the allogenic group (Vrieze et al., 2012). FMT of *Faecalibacterium prausnitzii*, a butyrate-producing bacteria, was also noted as a novel and effective strategy to treat diabetes (Ganesan et al., 2018). However, the application of FMT in metabolic diseases still faces challenges. Because the structure of the intestinal microbiome varies greatly among individuals and varies individually during the course of metabolic diseases, FMT is difficult to achieve using standardized microbiome composition to treat clinical symptoms in all patients. How to reduce the complexity of transplantation while establishing an effective and safe process for FMT preservation and preparation is an urgent issue for future research.

In contrast to FMT, probiotic products have a well-defined composition, and there have been many clinical trials exploring the effectiveness of probiotic therapies for metabolic diseases. Commonly used probiotics mainly include *Lactobacillus* and *Bifidobacterium*, and other probiotics, including *Akkermansia muciniphila*, *E. coli* Nissle 1917, are also being used in studies. Supplement of *Bifidobacterium* and *Lactobacillus* showed potential in improving fasting glucose and increasing insulin sensitivity in diabetes patients (Kijmanawat et al., 2019; Hasain et al., 2020). An earlier report showed bile salt hydrolase (BSH)-active *Lactobacillus reuteri* NCIMB 30242 administration increased volunteers’ bile acid pool and fibroblast growth factor 19 (FGF-19) levels but decreased their cholesterol levels (Jones et al., 2012; Martoni et al., 2015). In mild hyperglycemia and hyperlipidemia patients, *Bifidobacterium bifidum* TMC3115 supplement decreased plasma total cholesterol and low-density lipoprotein cholesterol (LDL-C) levels (Wang et al., 2019). Current studies are initiated to use more probiotic-probiotic or prebiotic-probiotic combinations to get the maximum effect of the intervention. Oral uptake of a six-probiotic mixture (*Lactobacillus acidophilus*, *L. rhamnosus*, *L. paracasei*, *Pediococcus pentosaceus*, *Bifidobacterium lactis*, and *B. breve*) in 12 weeks reduces the hepatic fat amount in NALFD patients (Ahn et al., 2019). Prebiotics and probiotics administration (*Bifidobacterium animalis* and 1.5 g of inulin yogurt) for 24 weeks also improves NAFLD and liver enzyme concentrations (Bakhshimoghaddam et al., 2018). However, as some studies reported failure outcomes of probiotics in controlling inflammation or preventing metabolic diseases, future probiotic research needs to overcome the limitations of existing studies, including the lack of standardization of clinical trial data, variability among strains, and individual differences in subjects, in order to improve microbiome interventions and efficacy.

Compared to an untargeted method such as FMT and probiotics, engineered microbes with specific genetic modifications could deliver more straight and significant impacts on host metabolism. A direct strategy is to produce bioactive proteins and metabolites to regulate host metabolism. Duan et al. designed an engineered *Lactobacillus gasseri* ATCC 33323 secreting human GLP-1 (1–37), an internal hormone regulating glucose metabolism. This inactive full-length form GLP-1 can stimulate intestinal epithelial cells into insulin-secreting cells. Administration of GLP-1 expressing *Lactobacillus* increased the insulin level in diabetic rats, leading to a reduction in blood glucose level (Duan et al., 2015). Some metabolic diseases which have a known mechanism of enzyme deficiency or malfunction like phenylketonuria and hyperammonemia, could be ameliorated directly by engineered microbes producing relative enzymes. Phenylketonuria is caused by the accumulation of phenylalanine (Phe) due to the dysfunctional enzyme in Phe metabolism, and the traditional treatment needs a protein-restricted diet to avoid high Phe intake. Isabella engineered *E. coli* Nissle 1917 to express LAAD and PAL in an anaerobic condition, which enabled the bacteria to convert Phe to phenylpyruvate (PP) and trans-cinnamate, respectively (Isabella et al., 2018). Deletion of the dapA gene, essential for cell wall biosynthesis and cell growth, could prevent the engineered bacteria from leaking into the environment. Administration of the final strain, SYNB1618, in mice and monkeys reduced their blood Phe concentrations and prevented Phe surge after oral Phe challenges. In another study, *E. coli* Nissle 1917 was engineered to consume intestinal NH_3_ for L-arginine biosynthesis in order to treat hyperammonemia (Kurtz et al., 2019). To enhance the NH_3_ consumption, arginine repressor ArgR was knocked out and a mutation was introduced to ArgA, the N-acetylglutamate synthase, to abolish the feedback inhibition. Furthermore, an essential thymidylate synthase gene thyA was deleted for biocontainment. The engineered strain named SYNB1020 notably lowered blood ammonia concentration and increased mice survival in a hyperammonemia mouse model. Producing bioactive metabolites also shows potential to treat metabolic diseases. *N*-acyl-phosphatidylethanolamines (NAPEs) are precursors of *N*-acyl-ethanolamines (NAEs), which are endogenous lipid satiety factors controlling food intake. Administration of high NAPEs-producing engineered *E. coli* Nissle 1917 (pNAPE-EcN) successfully improved the obesity of wild-type mice. Furthermore, administration of pNAPE-EcN ameliorated obesity and atherosclerosis lesion necrosis in a mice model of atherosclerosis (May-Zhang et al., 2019). These results demonstrate the potential of engineered microbes in treating metabolic diseases.

In the course of metabolic diseases treatment, monitoring metabolites concentration *in vivo* helps to judge the disease process in order to update the treatment plan in time. Several metabolite-specific sensor systems have been developed to detect the concentration of metabolites in the body to reflect metabolic or inflammatory status. For example, Daeffler et al. (Daeffler et al., 2017) identified a two-component system from marine *Shewanella* species sensing thiosulfate or tetrathionate, which are the biomarkers of inflammation. This sensor was used to drive a GFP reporter in *E. coli* Nissle 1917. This system was sensitive to both thiosulfate and tetrathionate *in vitro* with a good dose response. Using a similar strategy, Woo et al. (2020) constructed a nitrate sensing system and combined it with a thiosulfate sensor using a Boolean AND gate in an *E. coli* Nissle 1917 strain, which can thus respond to the coexistence of nitrate and thiosulfate to represent inflammation level. The key for metabolite monitoring is how to record and decode the data. Mimee et al. (2015) compared three methods for data recording and decoding. The first method was based on genome memory, where the concentration of the metabolite was converted to the inversion ratio of a specific DNA sequence by an integrase under the control of a metabolite-specific inducible promoter. The second method linked the expression level of NanoLuc luciferase to metabolite concentration, using luminescence as a readout. The third strategy also used luminescence strength as a readout. In this case, dCas9 was activated upon the detection of metabolites which degraded the mRNA of the NanoLuc reporter, thus reducing the luminescence level. Evaluation of these three methods *in vivo* were performed using *Bacteroides thetaiotaomicron* as a carrier, and the feces were collected every day for readouts. The results indicated that the genome memory readout was faster and more stable than the luminescence method, while further improvement of its orthogonality was still needed. In conclusion, engineered microbes for treatment of metabolic diseases is a promising method, and is expected to have more advanced construction strategies and to be validated in more metabolic diseases.

## The Microbiome and Tumors

**FIGURE 2:**
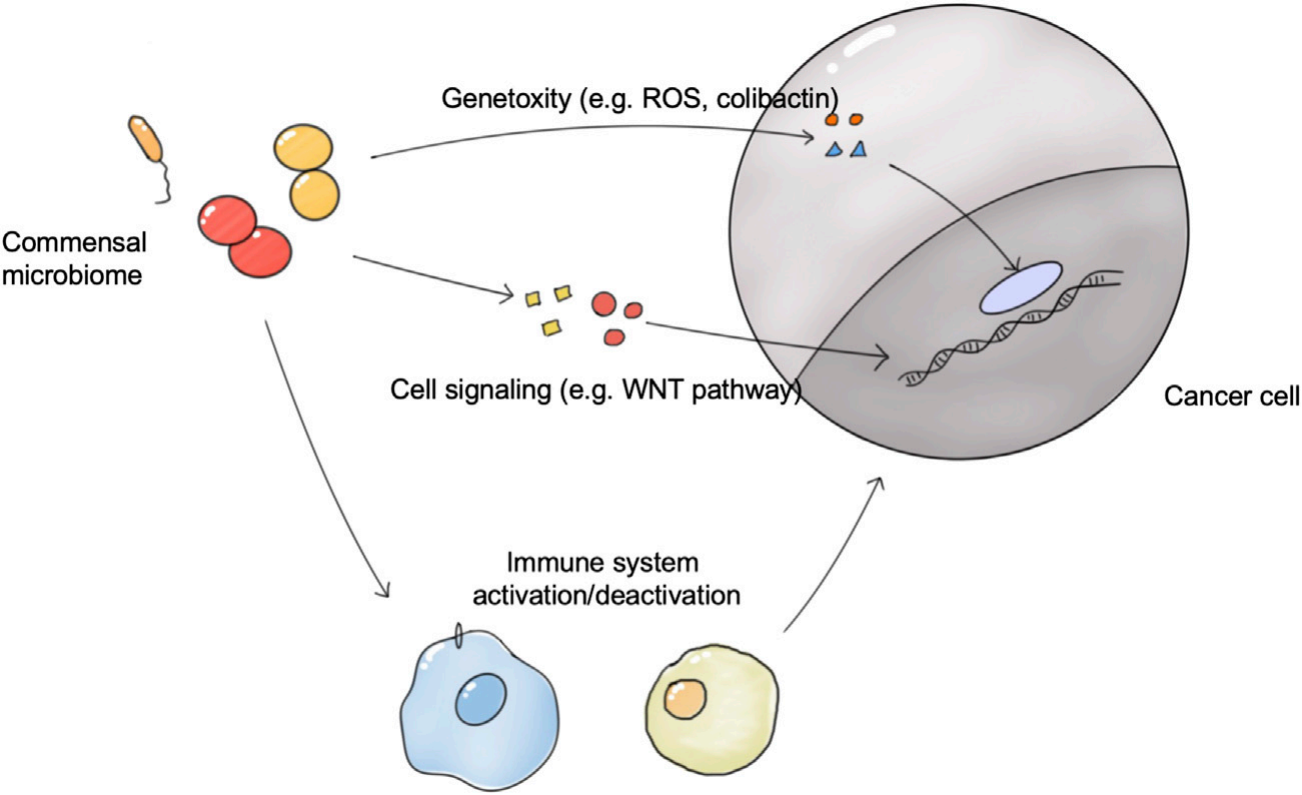
The connections between the commensal microbiome and tumors. The microbiome directly induces tumorigenesis via genotoxicity and the disrupted cell signaling pathway. The microbiome in organs and tumors influences the activation status of antitumor immune system responses, and thus affects the growth of tumors and tumor therapy outcomes.; Abbreviation: ROS, React oxygen species.

Microbes and tumors were linked for the first time when Coley observed and reported that bacterial infections can cause tumor regression (Coley, 1891). Many case studies and epidemiological studies have reported a strong relationship between the commensal microbiome and cancer development and therapeutic outcomes (Fletcher et al., 2018; Sepich-Poore et al., 2021). It is estimated that pathogen microbes drive 15–20% of cancer cases, and 30–35% of cases are driven by diet and environment factors, which are also closely related to the commensal microbiome (Bhatt et al., 2017). In 2019, the Cancer Microbiome Consortium first published an international expert consensus on the role of the microbiome in cancer. Experts confirmed the broad molecular mechanisms by which the human microbiome may be involved in the tumorigenesis, metastasis, and prognosis of cancer, including genotoxicity, inflammation, and immunity (Scott et al., 2019). In addition, the commensal microbiome influences the effectiveness of tumor treatment (Figure 2). Exploiting the properties of the microbiome at the time of tumor appearance is expected to have beneficial effects on tumor treatment.

### Association of the Microbiome and Tumor

Some microbiome species have been proven to be associated with tumorigenesis, providing a map of links between specific microbiome species and tumors (Borchmann, 2021; Dohlman et al., 2021). Of these, the gut microbiome was most frequently reported to be involved in tumorigenesis. For example, in colorectal cancer (CRC), *Bacteroides fragilis* could destroy the colonic mucosal protective layer by releasing toxins which led to tumorigenesis (Dejea et al., 2018). The accumulation of *F. nucleatum* has also been confirmed in multiple cohorts of CRC patients. In addition, dysbiosis is shown to be a causative factor of bladder, kidney, and prostate cancers (Liss et al., 2018; Markowski et al., 2019).

In the vagina microbiome, stable dominance of *Lactobacillus* is associated with a reduced risk of cancer of the female reproductive system. Cervical intraepithelial neoplasia (CIN) is a precancer state with abnormal cell growth. In high-risk HPV infection patients, the increase of *Lactobacillus* abundance and fungal diversity showed a negative correlation with CIN, while *Gardnerella vaginalis* and bacterial diversity showed a positive correlation with HPV infection (Usyk et al., 2020). In ovarian cancer patients, a similar decrease in the abundance of cervicovaginal *Lactobacillus* was observed. In particular, the abundance of cervicovaginal *Lactobacillus* was negatively correlated with the BRAC mutation significantly, which is a high-risk gene for ovarian cancer (Nene et al., 2019). Thus, reconstructing the vagina microbiome by *Lactobacillus* will be beneficial in avoiding HPV infection, cervical cancer, and ovarian cancer risk.

The oral microbiome has recently been found to correlate with a variety of cancers and can be used as a cancer prediction marker. There appears to be different compositions and diversity of the oral microbiome in different types of cancer. In CRC patients, the decrease of *Lachnospiraceae* is associated with higher CRC risk (Flemer et al., 2018). It is speculated that the protective effect of *Lachnospiraceae* is achieved via the inhibition on the proliferation and translocation to the gut of several oral pathogens (e.g., *F. nucleatum*, *Parvimonas micra*, *Peptostreptococcus stomatis*, and *Dialister pneumosintes*) and oral biofilm-forming bacteria (e.g., *Actinomyces*, *Hemophilus*, *Rothia*, *Streptococcus,* and *Veilonella* spp.). Fan et al. (2018) reported that the presence of oral pathogens *Porphyromonas gingivalis* and *Aggregatibacter actinomycetemcomitans* was positively correlated with pancreatic cancer risk, while Phylum Fusobacteria and its genus *Leptotrichia* in the oral microbiome were inversely correlated with pancreatic cancer risk. Hayes et al. (2018) reported that the risk of head and neck squamous cancer (HNSCC) is negatively correlated with *Corynebacterium* and *Kingella* abundance in the oral microbiome and the correlation was stronger in larynx cancer patients and in former smokers. Therefore, monitoring of the oral microbiome may serve as a simple cancer predictor (Flemer et al., 2018).

Recently, the presence of certain species and abundance of the microbiome within tumors has been demonstrated, and different tumors prefer different microbiome compositions. The seminal study by Ravid Straussman et al. showed that each type of tumor has a unique microbial composition and that some tumor tissues that are not in direct contact with the external environment, such as glioblastoma and bone tumors, have detectable bacterial presence, especially breast cancer (Nejman et al., 2020). The abundance and diversity of the microbiome of breast cancer in particular are high. The majority of bacteria within tumor tissues are localized intracellularly, with colonization in both tumor cells and immune cells. Lymphocytes contain more bacterial fragments, which may modulate the local immune response of the tumor. Bullman et al. (2017) observed that the microbiome in the primary colorectal environment and its derived distal liver metastasis tissue exhibited a similar microbiome composition, including *Fusobacterium* and its related microbiome bacteria *Bacteroides*, *Selenomonas*, and *Prevotella,* and indicated that the tumor microbiome may travel with tumor cells and colonize in the metastatic site. In addition, the use of antibiotics towards *Fusobacterium*-colonized patient-derived xenografts reduced tumor growth *in vivo*, suggesting that the intratumor *Fusobacterium* promotes tumor growth. However, there are only a few studies and it is not clear how the intratumor microbiome contributes to tumor development.

Currently, the mechanisms by which the microbiome affect cancer includes genotoxicity, inflammation, and immunity. The microbiome can influence the immune and inflammatory status of the host, similar to the effect of the microbiome on immunity and inflammation in metabolic diseases. For example, the disruption to gut microbiota homeostasis could generate a large number of inflammatory substances, such as some subtypes of bile acids, which were secreted and transported into the liver and induced an immune inflammatory response, eventually causing hepatocellular carcinoma genesis (Yu and Schwabe, 2017). Recently, Kalaora et al. (2021) reported a novel mechanism that the intratumoral microbiome presented tumor-specific antigens to activate the immune system. Intratumoral bacteria in 17 melanoma metastases derived 248 and 35 unique human leukocyte antigen class I and II (HLA-I and HLA-II) peptides which can be presented to activate immune system responses upon tumors. The genotoxic role played by the microbiome is a pressing issue in current research. Genotoxicity refers to direct or indirect damage to host DNA, which promotes cancer development. For example, *E. coli* strains containing the pks (polyketide synthase) pathogenicity island produces colibactin, a genotoxin which induces DNA damage (Buc et al., 2013). Besides, the microbiome could influence the cell signaling pathway in host cells, promoting tumorigenesis. Somatic mutations in TP53, most of which inactivate the tumor-suppressor function of P53, are the most abundant tumorigenesis mutations in humans (Freed-Pastor and Prives, 2012). In squamous cell carcinoma, a group of taxa including *Acidovorax* and *Klebsiella* were enriched more significantly in patients carrying the TP53 mutation, indicating that microbiome-genome factors played an important role in tumorigenesis (Greathouse et al., 2018). Recently, one important experiment illustrated microbiome-genome interactions on tumorigenesis. The group introduced a common human TP53 mutation into a mice model of intestinal cancer, which should enhance the tumor suppressor function of P53 by disrupting the WNT pathway. However, this tumor suppressor function was only observed in the proximal gut but not in the distal gut. Interestingly, microbiome removal in the distal gut restored the tumor suppressor function of mutate P53, indicating that the gut microbiome participated in the tumorigenesis of distal gut tumors. Metabolite screening found that gallic acid was responsible for the disruption of mutate P53 function, supported by the supplement of gallic acid which can abolish the tumor-suppressive effect of mutate P53 (Kadosh et al., 2020).

### The Microbiome and Cancer Treatment

The commensal microbiome could bi-directionally affect the outcome of immunotherapy (Sivan et al., 2015; Vetizou et al., 2015; Gopalakrishnan et al., 2018). The disturbance of the immune system is critical in tumor development, such as the disruption of immune checkpoints like programmed cell death protein 1/programmed cell death protein 1 ligand 1 (PD-1/PD-L1) or cytotoxic T lymphocyte-associated protein-4 (CTLA-4). The use of immune checkpoint inhibitors (ICIs) can induce a durable antitumor response in the body, however, the response to ICIs differs from patients, resulting in a total response rate close to 30%. Commensal microbiome composition has been reported to be correlated with the responsiveness of patients to ICIs. Enrichment of *Roseburia hominis* was associated with a responsive immune response to ICIs, while *Veillonella parvula* was associated with a non-responsive immune response to ICIs (Shaikh et al., 2021). Prediction based on patient microbiome composition showed a good accuracy on ICIs immune responsiveness. Peng et al. (2020) analyzed the gut microbiome of 74 patients with gastrointestinal cancers (including colorectal, gastric, and esophageal cancers) before and during their treatment with PD-1/PD-L1 monoclonal antibodies. Gut *Prevotella*/*Bacteroides* ratio, and the abundance of SCFAs-producing bacteria including *Eubacterium*, *Lactobacillus*, and *Streptococcus*, were positively associated with better clinical response. A predictive model based on bacterial taxa could accurately predict patient response to PD-1/PD-L1 monotherapy in the cohort of this study and in two other cohorts. On mechanism insights, the microbiome could activate the immune cell signaling pathway, and thus enhance immunotherapy outcomes. Oral administration of live LGG, thus increased the abundance of *Lactobacillus murinus* and *Bacteroides uniformis* in the gut, which activated dendritic cell (DC) IFN-β production via the cGAS/STING axis, and eventually resulted in CD8^+^ T cells activation and infiltration in tumors (Si et al., 2021).

The commensal microbiome could also affect the outcomes of chemotherapy. The chemotherapeutic drug oxaliplatin is relied on in the induction of ROS in tumors. In germ-free mice, the ROS production induced by oxaliplatin was lower than that in WT mice, suggesting that the antitumor effect of oxaliplatin is partly dependent on the commensal microbiome via the ROS pathway (Iida et al., 2013). Microbial-mediated metabolism of chemotherapeutic agents is an important factor affecting the efficacy of chemotherapy. Fluorouracil (5-FU) is the first-line therapeutic agent for colorectal cancer, however, there are large inter-individual differences in efficacy. Scott et al. established a traceable model of drug metabolism using *E. coli* and *C. elegans* to study the effect of the intestinal microbiome on the anticancer effect of fluorouracil. The results showed that ribonucleotide metabolism within the bacteria significantly influenced the action of fluorouracil, that the nucleoside diphosphate kinase ndk-1 regulated the bacterial deoxynucleotide pool and enhanced 5-FU-induced autophagy and cell death in host cells, and that vitamins B_6_ and B_9_ inhibited the action of fluorouracil through one-carbon unit metabolism (Scott et al., 2017). This illustrates the bidirectional potential of bacterial actions on chemotherapeutic agents to be targets of drug intervention to accurately modulate the effect of the microbiome on chemotherapeutic efficacy. The combination of CpG-oligonucleotide (ODN) and inhibitory interleukin-10 receptor antibody (anti-IL-10R) could induce tumor necrosis via a TNF-dependent pathway. In a mouse MC38 tumor model, Iida reported that depletion of the gut microbiota significantly reduced TNF activation and weakened the antitumor effects of ODN and anti-IL-10R. 16S sequencing of the gut microbiota revealed that the Gram-negative genus *Alistipes* and the Gram-positive genus *Ruminococcus* were positively correlated with TNF expression, whereas the Gram-positive genus (*Lactobacillus*) was negatively correlated. Reimplantation of *Alistipes shahii* into MC38 tumor-bearing mice could rescue TNF level and enhance immunotherapeutic effects (Iida et al., 2013).

Collectively, these results highlight the immune system regulatory function of the microbiome and the promise of microbiome therapy in combination with cancer therapy. Recently, Stephen et al. reported for the first time that intratumor fungi and bacteria play a completely different role during radiotherapy. The presence of intratumor bacteria is necessary for the antitumor immune response of the body after radiotherapy, whereas intratumor fungi suppress the antitumor immune response of the body after radiotherapy by binding to the receptor Dectin-1, which upregulates pro-tumor macrophages and downregulates antitumor T cells. This result suggests that excessive antibiotic use after radiotherapy can lead to the over proliferation of intratumor fungi, thus inhibiting the killing effect of radiotherapy (Shiao et al., 2021). This result emphasizes the important impact of the commensal microbiome on tumor therapy and suggests that future tumor treatment studies need to include the impact of the commensal microbiome more frequently in studies and interventions.

### Microbiome Intervention and Engineered Microbe Applications

It is not yet possible to directly eliminate cancer by intervening in the microbiome, so the current microbiome interventions aim mainly to enhance the effectiveness of cancer treatment or to reduce the toxicity and side effect of cancer treatment. FMT increases the levels of *Bifidobacterium longum* and *Faecalibacterium prausnitzii* in the gut of patients with PD-1 refractory melanoma, which were associated with effective immunotherapy responsiveness, and thus improves immunotherapy outcomes in some patients with a favorable safety profile (Davar et al., 2021). However, because cancer patients often have a disrupted or suppressed immune system and cancer treatment can damage the normal commensal microbiome and weaken the body’s normal immune barrier, FMT is riskier, and more research is needed to determine donor selection and operational steps for FMT in the cancer context. In contrast, the use of dietary or probiotic interventions for the microbiome may be safer and more effective, and a number of studies have been conducted (Panebianco et al., 2018; Zhang et al., 2019b; Lee et al., 2020). The fasting mimicking diet (FMD), which is a plant-based low amino-acid substitution diet, consisting of soups, broths, liquids, and tea, is reported to restore a heathy gut microbiome. In breast cancer patients, FMD significantly enhanced chemotherapy response, and also attenuated chemotherapy-induced T-cell DNA damage (de Groot et al., 2020). Probiotics mixture (including *Lactobacillus* and *Bifidobacterium strains*) were administrated for 4 consecutive weeks after CRC surgery. The ELISA result showed that pro-inflammatory cytokines TNF-α, IL-6, IL-10, IL-12, IL-17A, IL-17C, and IL-22 were significantly reduced in the probiotic group, facilitating patients’ recovery after cancer surgery (Zaharuddin et al., 2019). Thus, dietary interventions and oral probiotics can reshape the gut microbiome, improve immune response, reduce adverse effects, and hold promise as an important component of standardized cancer treatment.

Compared to non-targeted microbiome interventions, the modified engineered microbes are expected to have more direct tumor-killing and immunomodulatory effects (Zhou et al., 2018). Some studies have reported migration and colonization of bacteria into the tumor because of the hypoxia microenvironment of the tumor (Malmgren and Flanigan, 1955; Staedtke et al., 2015). This property becomes an important mechanism for the delivery of targeted tumor drugs by engineered microbes. Using this feature, *E. coli* Nissle 1917 or attenuated *Salmonella typhimurium* VNP20009 were designed for delivery of killer toxins like p53 and Tum-5 protein or DNase I into the tumor, resulting in tumor regression (Chen T et al., 2019; He et al., 2019). Furthermore, targeted intratumor delivery of a nanoantibody is a promising method of engineered microbes. In a mouse model with implanted A20 lymphoma cells, which was used to represent CD47-expressing tumors, Chowdhury et al. (2019) tested a reprogrammed *E. coli* (SLC-CD47) containing a synchronized lysis circuit (SLC) and a therapeutic effector, a CD47 antagonist nanobody. After intratumor or intravenous injection, SLC-CD47 significantly reduced the tumor volume and metastases and increased mouse survival rate. Surviving mice also showed tumor resistance when rechallenged by another tumor implantation. In 2020, Gurbatri et al. (2020) used the same synchronized lysis circuit to generate engineered *E. coli* Nissle 1917 carrying the PD-L1 nanobody (SLC:PD-L1nb) or CTLA-4 nanobody (SLC:CTLA-4nb) in a mouse A20 tumor model. The injection of engineered bacteria (SLC:PD-L1nb and SLC:CTLA-4nb) led to tumor regression and systematic activation of CD4^+^FOXP3^+^ T cells and CD8^+^ T cells. In poor immunogenic CT26 tumor models, another engineered bacteria (SLC:GM-CSF) expressing granulocyte-macrophage colony-stimulating factor (GM-CSF) were applied to enhance the antitumor effect of SLC:PD-L1nb and SLC:CTLA-4nb. The combination of three engineered bacteria strains effectively reduced tumor volume and increased survival. In addition to direct enrichment to tumor sites, engineered microbes can also remotely express relevant antigens or immunomodulatory substances, thereby enhancing tumor therapeutic effects (Chung et al., 2021; Griffin et al., 2021). In summary, an engineered microbe as a live platform to enhance immune response and tumor killers will become an important tool in cancer treatment.

## The Microbiome and Pathogen Infection

**FIGURE 3:**
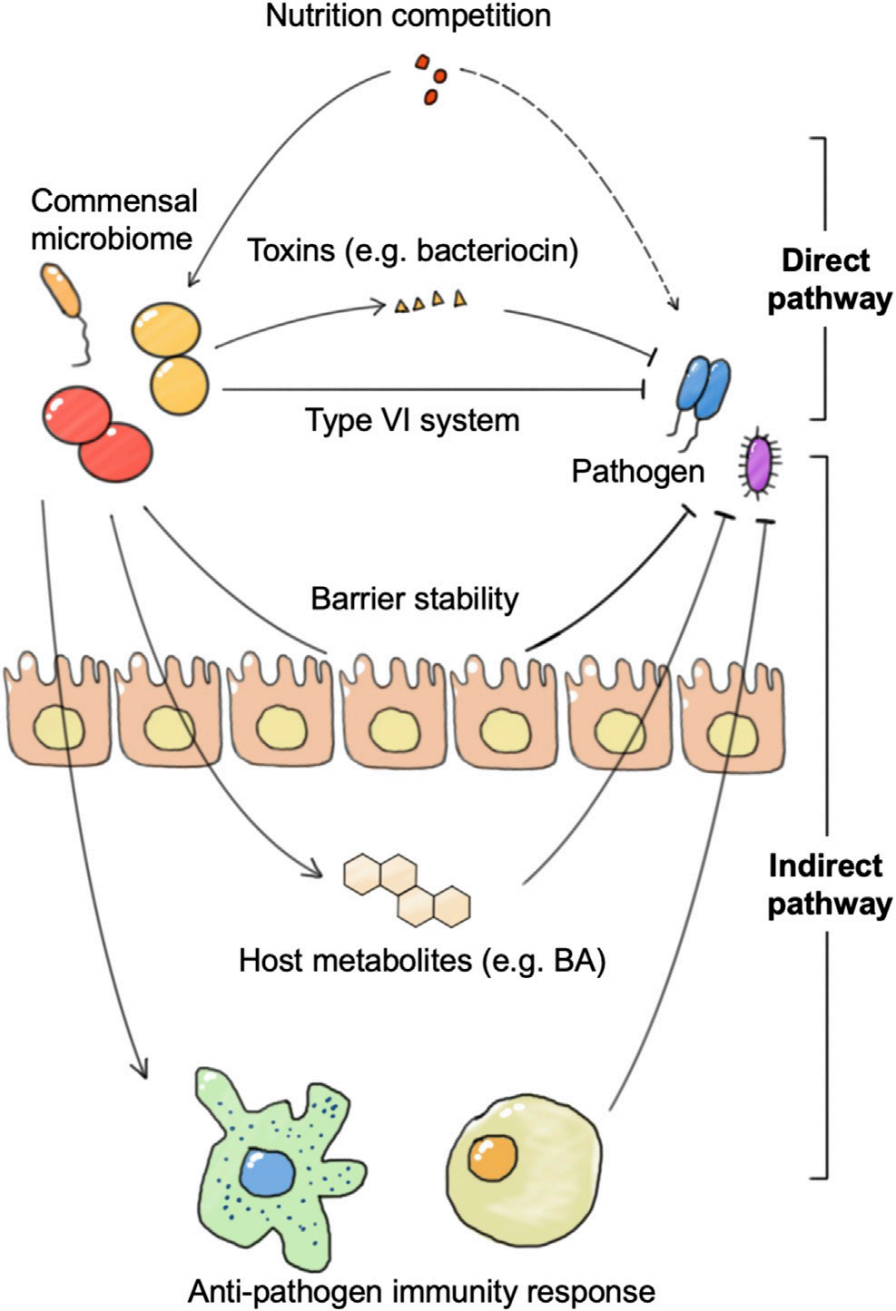
The connections between the commensal microbiome and pathogen. The commensal microbiome represents colonization resistance via direct and indirect pathways. The direct pathway includes nutrition utilization advantage against pathogens and direct killing of pathogens by toxins or the type VI secretion system. The indirect pathway refers to the activation of the host immune system or anti-pathogen metabolite production, and the enhancement of barrier stability.

The generally defined “healthy” commensal microbiome is regraded to be resistant to pathogen infection, which is called colonization resistance. Colonization resistance can be achieved through microbial competition for nutrients and the production of metabolites or bacteriocins, or indirectly through the induction of host immune responses (Figure 3). Microbiome intervention methods including FMT have shown good potential against several pathogen invasions, such as *Salmonella* enterica serovar *Typhimurium* and *Clostridioides difficile* (*C. difficile*). In anti-pathogen therapy, the application of engineered microbes could aid in the identification and monitoring of pathogens, as well as in the removal of pathogens.

### Colonization Resistance and Pathogen Infection

Disruption of the commensal microbiome including the gut microbiome, skin microbiome, and vaginal microbiome promotes pathogen infections. Strong relationships have been reported between dysbiosis and sequential sepsis with gut microbiome disorder (Prescott et al., 2015; Haak and Wiersinga, 2017). Analysis of bloodstream and the gut microbiome confirmed that the causative strains in some patients’ bloodstream infections are possibly from the gut microbiome, such as *Escherichia coli*, *Klebsiella pneumoniae, Pseudomonas aeruginosa,* and *Staphylococcus epidermidis* (Tamburini et al., 2018). Besides the gut microbiome, vaginal microbiome dysbiosis is also reported to promote sepsis. For example, vaginal microbiome dysbiosis characterized by *Lactobacillus* spp. depletion also exacerbated the risk of preterm pre-labor rupture of the fetal membranes and subsequent neonatal sepsis (Brown et al., 2018). On skin, various coagulase-negative *Staphylococci* (CoNS) isolated from the healthy skin microbiome could produce autoinducing peptides that inhibit *the Staphylococcus aureus* agr system and reduce PSMα production (Williams et al., 2019). Besides, CoNS species including *Staphylococcus epidermidis* and *Staphylococcus hominis* can also produce antimicrobial peptides which selectively kill *S. aureus*. Skin microbiome disorder promotes opportunistic pathogen *Staphylococcus aureus* proliferation, which secretes proteases and phenol-soluble modulin α (PSMα) through a quorum-sensing mechanism, damages the skin barrier, and promotes skin inflammation in mice. These results suggest that dysbiosis of the commensal microbiome contributes to the pathogenesis of pathogen infections.

The commensal microbiome achieves colonization resistance to pathogen infection in both direct and indirect mechanisms. The direct mechanism refers to the fact that the commensal microbiome achieves growth inhibition and clearance of pathogens directly without host participation, including mechanisms such as competition for nutrients and secretion of substances such as bacteriocins. For example, *B. thetaiotaomicron* consumes nutrition used by *C. rodentium*, which contributes to the competitive exclusion of the pathogenic bacteria from the intestine. *B. thetaiotaomicron* also releases fucose to suppress enterohaemorrhagic *E. coli* (EHEC) using sugar and prevents EHEC from competing for fucose with commensal *E. coli* (Bäumler and Sperandio, 2016). In addition to nutritional competition, there are many species of the commensal microbiome that can directly release lethal substances to remove pathogens or affect their growth and adhesion. *B. thuringiensis* secretes a bacteriocin that directly targets spore-forming Bacilli and Clostridia. A variety of *Bifidobacterium* spp. produce organic acids and peptides that impair growth and adhesion of pathogenic *E. coli* to enterocytes (Buffie and Pamer, 2013).

The indirect mechanism refers to the fact that the commensal microbiome achieves growth inhibition and clearance of pathogenic bacteria by activating the host’s immune response or stimulates host anti-microbial molecular production. Certain bacteria can increase phagocytosis of pathogenic bacteria by activating host DCs and macrophages. DCs and macrophages not only phagocytose pathogenic bacteria directly, but also activate downstream innate lymphoid cells and promote their secretion of anti-microbial cytokines. These cytokines further enhance epithelial expression of anti-microbial peptides. In addition to the innate immune system, adaptive immunity plays an important role in colonization resistance. Segmented filamentous bacteria (SFB) closely associate with the intestinal epithelium and enhance IgA production by B cells, serum amyloid A (SAA)-dependent T helper 17 (TH17) cell differentiation, pro-inflammatory cytokine production, and epithelial production of antimicrobial peptides (Kim et al., 2017). These processes confer protection against *Citrobacter rodentium*. There are also many bacteria, such as *B. thetaiotaomicron*, that do not depend on the immune system, but directly stimulate the intestinal epithelial cells to secrete a variety of anti-microbial peptides that directly limit the growth of pathogenic bacteria (Buffie and Pamer, 2013). In addition to this, some bacteria can resist pathogens by promoting the production of metabolites such as bile salts and short-chain fatty acids in the host. A recent study showed that *C. scindens* could convert primary to secondary bile salts to prevent *C. difficile* colitis (Pamer, 2016; Ducarmon et al., 2019). Even pathogenic bacteria *Klebsiella pneumonia* can train a host to enhance resistance to pathogens through bile acids (Stacy et al., 2021). The commensal microbiome can also achieve colonization resistance to viruses, which is more often achieved by activating the antiviral response of the immune system. In a vesicular stomatitis virus (VSV) infection model, *Bacteroides* glycolipids activated colonic DCs through the TLR4-TRIF signaling pathway, and thus stimulated IFN-β production in the gut and enhanced colonization resistance to viral infection.

### Microbiome Intervention and Engineered Microbes on Combating Pathogens

Currently, microbiome transplantation is already clinically applied for pathogen infections such as *Clostridioides difficile* infection (CDI). CDI is the most important risk factor contributing to antibiotic-associated diarrhea in hospital, and is prevalent in many developed countries. Recurrent *C. difficile* infection (rCDI), in the case of atypical and vancomycin resistance is difficult to treat, and first-line antibiotics therapy may induce the expansion of antibiotic-resistant organisms (AGOs) such as VRE (Al-Nassir et al., 2008; Deshpande et al., 2016). Kwak et al. (2020) reported that transplantation of RBX2660, an FMT drug, can significantly recover rCDI patients’ microbiome diversity and eliminate major AGOs. Besides, Jiang et al. (2018) confirmed that the effectiveness of oral lyophilized fecal microbiota and FMT by enema on CDI are similar. An exploratory study demonstrated the usage of vaginal microbiome transplantation to treat symptomatic, intractable, and recurrent bacterial vaginosis patients. Transplantation of a healthy volunteer microbiome remarkably improved patients’ symptoms and reconstructed a *Lactobacillus*-dominated vaginal microbiome, and relieved patients’ bacterial vaginosis long-term (Lev-Sagie et al., 2019). This implies the diversity of microbiome transplantation forms that can help expand the application of microbiome transplantation. In addition, clinical studies of FMT for more difficult multidrug-resistant (MDR) pathogen infections, such as extended spectrum β-lactamase (ESBL)-producing and carbapenemase-producing *Enterobacteriaceae*, vancomycin-resistant *Enterococci* (VRE), or methicillin-resistant *Staphylococcus aureus*, are likewise being conducted, and several clinical cases reported their efficacy and safety (Freedman and Eppes, 2014; Singh et al., 2014; Crum-Cianflone et al., 2015; Stripling et al., 2015; Wei et al., 2015).

The use of probiotics could also treat pathogen infections. Probiotics usage can be considered as a microbiome transplantation of known microbiome composition, so it may be more applicable to those infections with clear probiotic-pathogen interactions. For example, the vaginal microbiome is an important commensal microbiome in women, and participates in vaginal homeostasis. It is known that a *Lactobacillus*-dominated vaginal microbiome is beneficial to prevent vaginal infections. In HPV-infected women, reconstruction of the vaginal microbiome via *Lactobacillus rhamnosus* BMX 54 implementation aids the clearance of HPV-infection, and a long-term (6 months) probiotic group showed a higher percentage of clearance of HPV infection (Palma et al., 2018) compared to the short-term (3 months) probiotic group (Palma et al., 2018). In ventilator-associated pneumonia (VAP) patients, the pathogenic enteric bacteria aspirated from the oropharynx is regarded the main etiology. VAP patients treated with probiotic *Lactobacillus plantarum* 299 (Lp299) to reconstruct gut microbiota exhibits the same effectiveness as antibiotic (chlorhexidine) treatment, implying that the probiotic usage may be an alternative way to control chronic infection (Klarin et al., 2018). Combining probiotics and prebiotics treatment (*Bifidobacterium breve* strain Yakult, *Lactobacillus casei* strain Shirota, and galactooligosaccharides) has also been reported to be effective in preventing sepsis patients from enteritis or VAP (Shimizu et al., 2018). These studies suggest that probiotics could perform as a microbiome regulator to prevent or treat pathogen infection. Although the current range of applications is still narrow, as the understanding of microbiome-pathogen interaction improves, more probiotic drugs will be available in the future as primary or adjunctive agents for a wide range of infections.

With increased understanding of pathogen-host interactions, engineered microbes can be used as targeted tools in pathogen infections. First, engineered microbes can be used *in vivo* for differential diagnosis or detection of pathogen infections. Unlike traditional time-consuming diagnostic approaches that require culture, engineered microbes can rapidly detect the infecting pathogen species by carrying sensors that sense pathogen characteristics and thus provide guidance for subsequent treatment. The work of Mao et al. (2018) provides a proven paradigm for engineering microbes to aid in the diagnosis of pathogens. *Vibrio cholerae* produces the characteristic quorum-sensing molecule cholera autoinducer 1 (CAI-1). Mao integrated a sensor that senses CAI-1 with a downstream β-lactamase into *Lactococcus lactis* as an engineered microbe that senses *V. cholerae*. When gavaged to mice, engineered *Lactococcus lactis* sensed CAI-1 and initiated β-lactamase expression. Mice feces containing β-lactamase catabolized nitrocefin and produced a color change, thus suggesting *Vibrio cholerae* infection. This study illustrates the feasibility of engineered microbes in pathogen diagnosis and suggests its effectiveness and simplicity.

Second, engineered microbes can directly inhibit or kill pathogens by enhancing colonization resistance mechanisms of commensal microbes against pathogens. Virulence factor is an important mechanism of pathogen infection, interfering with host physiological activity through the secretory system and promoting pathogen colonization. One study used virulence factor competition to prevent *Listeria monocytogenes* infection (Drolia et al., 2020). *L. monocytogenes* invasion is initiated by the expression of Listeria adhesion protein on its surface, which binds to heat shock protein 60 of host cells and disturbs the epithelial barrier. Nonpathogenic *Listeria innocua* has similar LAP but lack of secretion and surface exhibition, and thus behaves as a nonpathogen. Engineered probiotic *Lactobacillus casei* expressing *L. monocytogenes* or *Listeria innocua* origin LAP was used to compete for Hsp60 binding with *L. monocytogenes*. *The in vivo* result confirmed that engineered probiotic *Lactobacillus casei* significantly prevented *L. monocytogenes* infection, protected the intestinal barrier and homeostasis, and regulated immune responses. Activating the host’s own anti-pathogen mechanism can also be an effective strategy for the construction of engineered microbes. Leonard et al. (2020) engineered a microbe to express double-stranded RNA (dsRNA), thus triggering the host’s own RNA interference (RNAi) system. In infected models of bees, engineered commensal microbiota *Snodgrassella alvi* successfully eliminated deformed wing virus and parasitic *Varroa* mites, suggesting that engineered microbes can also treat pathogen infections besides bacteria. Fungi are more difficult to modify than bacteria, and their physiological activities are more complex; therefore, there are very few reports of medically engineered fungi. Chen et al. (2020) modified intestinal dwelling probiotic *Saccharomyces boulardii* expressing monoclonal antibodies against TcdA and TcdB, the major virulence factors of *Clostridioides difficile*, and demonstrated that engineered *S. boulardii* administration could protect mice from first or rCDI, and therefore can be used as an alternative way to FMT to treat CDI.

Combining pathogen sensing and killing becomes a trend in engineered microbes to defeat pathogens. For example, *Pseudomonas aeruginosa* usually invades neutropenic or immunocompromised patients. Saeidi et al. (2011) engineered a probiotic *E. coli* Nissle 1917 strain SED to first detect *P. aeruginosa* by its autoinducer N-acyl homoserine lactone (AHL) and then undergo self-lysis to release an anti-*P. aeruginosa* toxin, pyocin S5, to kill the pathogen (Saeidi et al., 2011). Further modifications, including the generation of an auxotroph complement and the expression of an anti-biofilm enzyme dispersin B (DspB) stabilized the genetic circuits and enhanced pathogen elimination. When administered in mice, the enhanced strain significantly decreased *P. aeruginosa* colonization and reduced its infection (Hwang et al., 2017). This combination of pathogen detection and killing microbes can be a more intelligent and simple strategy for fighting complex infections.

## Discussion

The commensal microbiome plays a different role in promoting or inhibiting disease processes such as metabolic diseases, cancer, and pathogenic invasion. Due to the extensive involvement of the commensal microbiome in the maintenance of homeostasis in the human body, microbiome intervention has become an emerging direction in disease treatment with high potential for clinical application. While traditional methods of microbiome intervention, including antibiotic treatment and fecal transplantation, have already been used in certain diseases, engineered microbes have the potential to be the next-generation probiotics due to their precision and versatility in regulating host physiological activities. In addition, engineered microbes can sense and record certain signals inside the body to reflect host health status (Mays and Nair, 2018; Riglar and Silver, 2018).

However, engineered microbes as next-generation probiotics still face many challenges due to the complexity of symbiotic microbial-host interactions. The first challenge in designing microbiomes is the lack of accurate and extensive understanding of host-microbiome interactions, which prevents us from precisely evaluating the growth of engineered microbes *in vivo* or the influence from the host and other symbiotic microbes to the expression of specific genetic circuits, making it difficult to predict the practical impact of the engineered microbes on the host. For example, SYNB1020 (NCT03447730), an engineered microbe drug which had success in an animal model and preclinical trials, did not achieve the expected results in phase I clinical trials, possibly due to the complexity of host-microbiome interactions where the metabolic protective function of the engineered microbe is counteracted by feedback inhibition from the host or other commensal microorganisms. The second challenge is the safety concerns on the engineered microbiome. Researchers have reported that horizontal gene transfer occurs frequently in the gut microbiome and more frequently in industrialized populations (Groussin et al., 2021). Meanwhile, to guarantee that the introduced engineered microbe is harmless to humans requires better strain construction strategies and careful preclinical studies. The third challenge is that we need new tools and strategies to engineer microbes and to introduce gene circuits *in vivo* and *in vitro*. Current gene editing tools often experience a high risk of off-targeting, and the efficiency of gene editing varies widely across microbial species. In addition, we need more synthetic biology parts that can sense a wider variety of signals, such as photosensitive promoters, to expand our means of *in vivo* manipulation of engineered microbes. In addition, most of the feedback systems are nonlinear and duration-dependent. To better detect and respond to changes in the hosts, genetic elements with a wider linear range are needed.
