# Supplementary material for: Human Microbiome and Its Medical Applications
Source: Front Mol Biosci. 2022 Jan 13;8:703585. doi: 10.3389/fmolb.2021.703585 (PMC8793671; doi:10.3389/fmolb.2021.703585)
Supplement: Supplementary file 1 [file Table1.docx]

**Supplementary Table 1**: Selected clinical RCTs using engineered microbes.

| Species | Strain | Engineering | Phases | Disease/conditions | NCT number | Enrollment | Status | Administration route |
| --- | --- | --- | --- | --- | --- | --- | --- | --- |
| *Lactococcus lactis* | AG011 | IL10 expression | I/II | Moderately active Ulcerative Colitis | NCT00729872 | 60 | completed | oral and enema |
|  | AG013 | Trefoil Factor 1 expression | IB | Oral mucositis | NCT00938080 | 25 | completed | oral |
| *Escherichia coli* Nissle 1917 | SYNB1020-CP-001 | Arginine feedback resistant N-acetylglutamate synthase | I | Hyper-ammonemia | NCT03179878 | 52 | completed | oral |
|  | SYNB1020-CP-002 | Study of Safety, Tolerability and Pharmacodynamics | I/II | Cirrhosis | NCT03447730 | 23 | terminated | oral |
|  | SYNB1618/1934 | Efficacy and Safety ， Phenylketonuria | II | Phenylketonuria | NCT04534842 | 24 | recruiting | oral |
|  | SYNB1618 | Safety and Tolerability， Phenylketonuria | I/II | Phenylketonuria/Healthy | NCT03516487 | 70 | completed | oral |
|  | SYNB1891 | Safety and Tolerability of SYNB1891 | I | Metastatic Solid Neoplasm/Lymphoma | NCT04167137 | 70 | recruiting | intravenous |
|  | SYNB1934 | Safety and Tolerability of SYNB1934 | I | Healthy Volunteers | NCT04984525 | 106 | recruiting | oral |
|  | SYNB8802 | Safety and Tolerability ，Enteric Hyperoxaluria | I | Healthy Volunteers | NCT04629170 | 65 | recruiting | oral |
| *Listeria monocytogenes* | BMB72 | Live-attenuated strain (ΔactA/ΔplcB), influenza A nucleoprotein expression | I | Healthy volunteers | NCT01311817 | 8 | completed | transcutaneous |
|  | ADXS11-001 | Live-attenuated strain, HPV-16-E7 expression | I | Human Papilloma Virus-related oropharyngeal cancer | NCT01598792 | 2 | terminated | intravenous |
|  |  |  | II | Cervical Intraepithelial Neoplasia | NCT01116245 | 81 | terminated | intravenous |
|  |  |  | II | Persistent or recurrent cervical cancer | NCT01266460 | 54 | completed | intravenous |
|  |  |  | I/II | (HPV+)-related cervical cancer | NCT02164461 | 25 | completed | intravenous |
|  |  |  | I/II | Anal cancer | NCT01671488 | 11 | completed | intravenous |
|  |  |  | II | Anal or rectal cancer | NCT02399813 | 51 | completed | intravenous |
|  |  |  | I/II | Cervical, head and Neck cancer (HPV+) | NCT02291055 | 66 | Active，Not recruiting | intravenous |
|  |  |  | II | Human Papilloma Virus-related oropharyngeal cancer | NCT02002182 | 15 | Active，not recruiting | intravenous |
|  |  |  | III | Human Papilloma Virus-related cervical cancer | NCT02853604 | 450 | Active，not recruiting | intravenous |
|  |  |  | II | Carcinoma, non-small-cell lung cancer (HPV+) | NCT02531854 | 124 | Unknown | intravenous |
|  | ADXS31-142 | Live-attenuated strain, PSA expression | I/II | Prostate cancer | NCT02325557 | 51 | Active，not recruiting | intravenous |
|  | ADXS31-164 | Live-attenuated strain, HER2 expression | I/II | HER2 expressing solid tumors | NCT02386501 | 12 | completed | intravenous |
|  | CRS-100 | Live-attenuated strain, mesothelin expression | I | Carcinoma and liver metastases | NCT00327652 | 9 | completed | intravenous |
|  | ANZ-100/ CRS-207 | Live-attenuated strain, mesothelin expression | I | Advanced solid tumors | NCT00585845 | 17 | terminated | intravenous |
|  |  |  | II | Metastatic pancreatic cancer | NCT01417000 | 93 | completed | intravenous |
|  |  |  | IIB | Greater Metastatic Pancreatic Cancer | NCT02004262 | 303 | completed | intravenous |
|  |  |  | I/II | Platinum-resistant ovarian, fallopian, or peritoneal cancer | NCT02575807 | 35 | terminated | intravenous |
|  |  |  | IB | Malignant pleural mesothelioma | NCT01675765 | 60 | completed | intravenous |
|  |  |  | II | Previously-treated metastatic adenocarcinoma of the pancreas | NCT02243371 | 93 | completed | intravenous |
|  |  |  | II | Malignant pleural mesothelioma | NCT03175172 | 10 | terminated | intravenous |
|  |  |  | II | Gastric, gastroesophageal junction or esophageal cancers | NCT03122548 | 5 | terminated | intravenous |
|  |  |  | II | Pancreatic cancer | NCT03190265 | 63 | recruiting | intravenous |
|  |  |  | II | Metastatic pancreatic adenocarcinoma | NCT03006302 | 44 | recruiting | intravenous |
|  | ADU-623 | Live-attenuated strain, EGFRvIII and NY-ESO-1 expression | I | Astrocytic Tumors; Glioblastoma Multiforme; Anaplastic Astrocytoma; Brain Tumor | NCT01967758 | 11 | competed | intravenous |
|  | JNJ-64041809 (ADU-741) | Live-attenuated strain, multiple cancer antigen expression | I | Metastatic castration-resistant prostate cancer | NCT02625857 | 26 | completed | intravenous |
|  | JNJ-64041757 (ADU-214) | Live-attenuated strain, mesothelin and EGFRvIII expression | I | Non-small cell lung cancer | NCT02592967 | 18 | terminated | intravenous |
|  | pLADD | Live-attenuated strain, patient specific neoantigen expression | I | Metastatic colorectal cancer | NCT03189030 | 28 | terminated | intravenous |
| *Salmonella* Typhimurium | x4550 | Live-attenuated strain, IL-2 expression | I | Cancer of the Liver | NCT01099631 | 22 | completed | oral |
|  | VNP20009 | examine the safety and toxicities | I | Cancer，Neoplasm Metastasis | NCT00004988 | 45 | completed | intravenous |
|  | CVD908ssb-TXSVN | tumor-associated antigens expression | I | Multiple Myeloma | NCT03762291 | 24 | recruiting | oral |
| *Bifidobacterium longum* | APS001F | CD enzyme convert the 5-FC into 5-FU | I/II | Tumors，Neoplasms | NCT01562626 | 75 | suspended | intravenous |
| *Clostridium* | Clostridium novyi-NT spores | The host immune and inflammatory response | I | Solid Tumor Malignancies  Malignancies | NCT01924689 | 24 | completed | intravenous |
